# The Archetypal Gamma-Core Motif of Antimicrobial Cys-Rich Peptides Inhibits H^+^-ATPases in Target Pathogens

**DOI:** 10.3390/ijms25179672

**Published:** 2024-09-06

**Authors:** María T. Andrés, Nannette Y. Yount, Maikel Acosta-Zaldívar, Michael R. Yeaman, José F. Fierro

**Affiliations:** 1 Laboratory of Oral Microbiology (LMO), Clinical University of Odontology (CLUO), University of Oviedo, 33006 Oviedo, Asturias, Spain; andresmaria@uniovi.es (M.T.A.);; 2 Instituto de Investigación Sanitaria del Principado de Asturias (ISPA), 33006 Oviedo, Asturias, Spain; 3 SamerLabs SL, Asturias Technology Park, 33428 Llanera, Asturias, Spain; 4 Divisions of Molecular Medicine and Infectious Diseases, Department of Medicine, Los Angeles County (LAC)—Harbor University of California—Los Angeles (UCLA) Medical Center, Torrance, CA 90502, USAmryeaman@ucla.edu (M.R.Y.); 5 Institute for Infection & Immunity, Lundquist Institute for Biomedical Innovation at Harbor UCLA, Torrance, CA 90502, USA; 6 Department of Medicine, David Geffen School of Medicine at UCLA, Los Angeles, CA 90095, USA; 7 Department of Functional Biology (Microbiology), Faculty of Medicine, University of Oviedo, 33006 Oviedo, Asturias, Spain

**Keywords:** gamma-core motif, H^+^-ATPase, ATPase inhibitor, lactoferrin, antimicrobial motif, antimicrobial peptide, antimicrobial mechanism of action, innate immunity, host defense

## Abstract

Human lactoferrin (hLf) is an innate host defense protein that inhibits microbial H^+^-ATPases. This protein includes an ancestral structural motif (i.e., γ-core motif) intimately associated with the antimicrobial activity of many natural Cys-rich peptides. Peptides containing a complete γ-core motif from hLf or other phylogenetically diverse antimicrobial peptides (i.e., afnA, SolyC, PA1b, *Pv*D_1_, thanatin) showed microbicidal activity with similar features to those previously reported for hLf and defensins. Common mechanistic characteristics included (1) cell death independent of plasma membrane (PM) lysis, (2) loss of intracellular K^+^ (mediated by Tok1p K^+^ channels in yeast), (3) inhibition of microbicidal activity by high extracellular K^+^, (4) influence of cellular respiration on microbicidal activity, (5) involvement of mitochondrial ATP synthase in yeast cell death processes, and (6) increment of intracellular ATP. Similar features were also observed with the BM2 peptide, a fungal PM H^+^-ATPase inhibitor. Collectively, these findings suggest host defense peptides containing a homologous γ-core motif inhibit PM H^+^-ATPases. Based on this discovery, we propose that the γ-core motif is an archetypal effector involved in the inhibition of PM H^+^-ATPases across kingdoms of life and contributes to the in vitro microbicidal activity of Cys-rich antimicrobial peptides.

## 1. Introduction

The transferrin family of proteins comprises a multifunctional group of homologous iron-binding glycoproteins (i.e., transferrin, lactoferrin, ovotransferrin, etc.) found in various cells and fluids of animals along the evolutionary continuum [1]. The ability of these monomeric proteins (76–81 kDa) to bind iron reversibly was initially associated with their antimicrobial activity. The first reported antimicrobial function for lactoferrin and transferrin was the deprivation of extracellular iron to potential pathogens, and by means of this indirect defensive activity, they were recognized as elements of innate immunity (reviewed in [2]). Subsequently, the antimicrobial activity of lactoferrin was shown to be independent of its iron saturation state [3,4,5,6], and the idea that this protein causes permeabilization of the plasma membrane was introduced, which has been experimentally refuted [7,8,9,10].

We previously discovered that human lactoferrin blocks microbial plasma membrane (PM) H^+^-ATPases, disturbing cellular ion homeostasis resulting in cell death in vitro [9,10,11,12]. Further, a similar antagonistic activity of bovine lactoferrin on vacuolar H^+^-ATPases (plasmalemmal V-ATPase) of yeast and tumor cells was reported [13,14], revealing important aspects of its target interactions (reviewed in [15]). To our knowledge, this antimicrobial mechanism of action has not been described for other effector molecules of the innate immune system. This phenomenon prompted us to explore structures of human lactoferrin that account for microbial H^+^-ATPase inhibition as a goal that could be translational for future novel anti-infective strategies and applications.

Among the most studied lactoferrin-derived antimicrobial peptides are those known as lactoferricin (Lfcin) and lactoferrampin [16,17]. It has been suggested that their corresponding sequences in lactoferrin are involved in the antimicrobial activity of the lactoferrin molecule. However, this idea does not fully explain why their mechanism of action in vitro clearly differs with respect to that observed for lactoferrin: the peptides lactoferricin and lactoferrampin disturb the integrity of target microbial plasma membranes [17,18,19,20], whereas the non-permeabilizing lactoferrin blocks H^+^-ATPases [9,10,11,14].

We previously reported the presence of the structure called the gamma-core (γ-core) motif within the human lactoferrin molecule (691 amino acids) and most of the transferrin family of proteins [21,22,23]. The γ-core motif is a three-dimensional (3D) structure, discovered by Yount and Yeaman [24], characterized by two anti-parallel *β*-strands connected by a hairpin loop. The formula γ-core motif ranges from 8 to 18 amino acid residues and contains archetypal patterns including (a) a conserved triad of residues (Gly-X-Cys, dextromeric isoforms; Cys-X-Gly, levomeric isoforms), (b) cysteine connectivity patterns, (c) net cationic charge, and (d) amphipatic stereogeometry. This structural pattern is observed in many natural cysteine-rich antimicrobial peptides (Cys-rich AMPs) representing a unifying structural signature intimately associated with antimicrobial activity [24]. The γ-core motif has been conserved during an evolutionary span of at least 2.6 billion years in some Cys-rich AMPs and proteins of bacterial, fungal, plant, and animal origin. This high degree of conservation suggests a crucial role in host defense functions of unicellular and multicellular organisms [24,25]. The two γ-core motifs identified by us in the transferrin family (i.e., N-lobe: C-(X_2_)-CXG-(X_1–7_)-C; C-lobe: C-(X_2_)-CXG-(X_3–14_)-C) exhibited antimicrobial activity and were the first found in large antimicrobial proteins, showing that human defensive proteins surprisingly include this ancient structure [21,22,23]. Subsequently, it was shown that the γ-core motif of human beta defensin-3 (hBD-3) recapitulates the antimicrobial activity of the full-length molecule [26]. The presence of γ-core motifs in different groups of antimicrobial proteins and peptides of the same animal species (e.g., human lactoferrin and hBD-3) underlines the key biological significance of this archetypal structural motif in innate host defense. However, the exact biological role of this ancestral structure in the antimicrobial activity of Cys-rich AMPs has not been fully elucidated to date.

The antimicrobial and multidimensional amino acid sequence that includes the levomeric isoform γ-core motif of human lactoferrin was previously synthesized and the resulting 31-amino acid peptide, called kaliocin-1 (UniProtKB accession number P02788|171-201; kaliocin-1), showed bactericidal and fungicidal activity in vitro [21,22]. Interestingly, this peptide also showed a similar mode of action to the human lactoferrin molecule, and common features associated with its antimicrobial activity have been reported previously [8,21,22,27] and can be summarized as follows: (a) the absence of either plasma membrane permeabilization, pore formation and/or membranolytic effects on microbial cells; (b) cation sensitive microbicidal (cidal) activity; (c) dysregulation of intracellular potassium ion flux (K^+^ efflux); and (d) dissipation of the transmembrane electrical potential (Δ*φ*). In addition, the essential role of mitochondrial ATP synthase and the influence of cellular respiration on the fungicidal activity of human lactoferrin, mediated by an apoptotic-like process, have been reported [10,12]. All of the above features induced by kaliocin-1 are clearly different from those reported for most known antimicrobial peptides [28], with some of these features constituting known events of a regulated cell death process [29]. On the other hand, this structure–activity relationship contrasts sharply with the membrane-disrupting effects described for other lactoferrin-derived peptides such as lactoferricins and lactoferrampin [17,18,19,20]. The similar mode of action of human lactoferrin and kaliocin-1 peptide correlated well with the presence of the γ-core motif in both molecules. Furthermore, this Cys-rich sequence includes amino acid patterns (i.e., *Rana*-box/insect-box loop structures) associated with the antimicrobial activity of other more evolutionarily distant natural defensive peptides [30]. For all of these reasons, we previously hypothesized that the γ-core motif of human lactoferrin is involved in the direct antimicrobial effect of this immunoprotein [21,22,23]. If so, this finding could provide an understanding of the structure–function relationships of the γ-core motif in the inhibition of microbial H^+^-ATPase. This biological function could putatively be extended to a wide range of other antimicrobial peptides/proteins containing this archetypal molecular structure. These hypothetical considerations prompted us to determine whether the γ-core motif is involved in the inhibition of microbial H^+^-ATPases.

For this purpose, the yeast *Candida albicans* and the Gram-negative bacterium *Pseudomonas aeruginosa*, both of which are aerobic microorganisms and important human opportunistic pathogens, were exposed to kaliocin-1 and selected kaliocin-1-derived peptides (Kdp) to evaluate their microbicidal efficacy and mechanisms. The structure–activity relationships associated with cell death and the influence of extracellular conditions were compared to those previously described for human lactoferrin and BM2 peptide, two PM H^+^-ATPase inhibitors [9,10,31]. Finally, we tested other Cys-rich AMPs or their derivate fragments, containing complete γ-core motifs, to evaluate their possible inhibitory activity on microbial H^+^-ATPases.

## 2. Results

### 2.1. Antimicrobial Activity of hLf γ-Core-Containing Peptides

To determine if the γ-core sequence of human lactoferrin (hLf) is directly involved in the antimicrobial activity of this protein, we compared the microbicidal activity of kaliocin-1 (Kal-1) and other five shorter derived peptides including variants in which one cysteine was exchanged by glycine (Table 1).

#### 2.1.1. Microbicidal Efficacy

**Figure 1:**
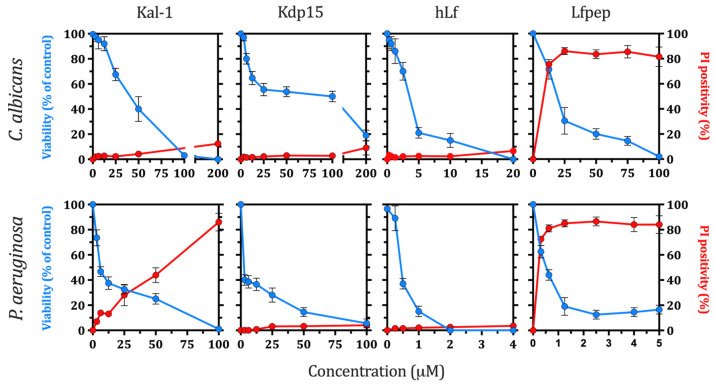
Cell viability and membrane permeabilization assays. *C. albicans* or *P. aeruginosa* (10^6^ cells/mL) were incubated (90 min, 37 °C) with each peptide/protein at the indicated concentrations. Aliquots were plated on SDA or TSB, and colonies were counted after 24–48 h (*C. albicans*) or 24 h (*P. aeruginosa*). At each point, samples were stained with propidium iodide (PI) and intracellular PI incorporation, indicative of membrane permeabilization, was quantified by flow cytometry. The relative cell viability (blue lines) and cellular permeabilization (red lines) were calculated as the percentage of untreated cells. Human lactoferrin (hLf) and the peptide Lfpep were used as negative and positive controls of cellular permeabilization, respectively. The results are the means of at least three independent experiments, and error bars represent standard deviations (±SD).

First, we tested the antimicrobial activity of Kal-1 and a shorter Kal-1-derived peptide (Kdp15), both containing the complete γ-core signature of human lactoferrin, against *C. albicans* and *P. aeruginosa*. Microbicidal (cidal) activity was determined after 90 min of incubation (37 °C; pH 7.4) with a range of concentrations of each hLf-derived peptide. The hLf γ-core-containing peptides were microbicidal in a concentration-dependent way (Figure 1). Kal-1 and Kdp15 showed a lower microbicidal efficacy as compared with hLf, and both peptides were more pseudomonacidal than candidacidal. The IC_50_ estimated for Kal-1 and Kdp15 against *C. albicans* was 40 μM and 100 μM, respectively. These values were approximately 13- and 33-fold greater than the IC_50_ calculated for the native protein hLf (3 μM). Under similar experimental conditions, the IC_50_ calculated against *P. aeruginosa* was 7.5 μM (Kal-1) and 3 μM (Kdp15), respectively. hLf and Lfpep were used in control assays as they exhibit microbicidal activity mediated by the inhibition of PM H^+^-ATPase or PM permeabilization, respectively. Lfpep is an antimicrobial hLf-derived peptide without a γ-core motif (Table 1) that permeabilizes bacterial and fungal cytoplasmic membranes [21,22]. Lfpep was the most active hLf-derived peptide showing the lowest IC_50_ for *C. albicans* (20 μM) and *P. aeruginosa* (0.5 μM). Unless otherwise stated, the concentration of each peptide able to kill 50% of cells (IC_50_) was selected for further assays, and the previous reported microbicidal concentrations of hLf against *C. albicans* (5 μM) and *P. aeruginosa* (1 μM) or the peptides Lfpep (50 μM) and BM2 (0.25 μM) were used in control assays [9,10,21,22,32]

#### 2.1.2. Cytoplasmic Membrane Permeabilization

To determine whether Kal-1 and Kdp15 exert a permeabilizing effect on fungal or bacterial membranes, the cellular influx of the fluorescent nucleic-acid-binding dye propidium iodide (PI) was assessed. As PI itself cannot permeate through an intact phospholipid bilayer, only permeabilized cells take up PI, resulting in DNA intercalation and fluorescence. PI fluorescent cells were not significantly increased after incubation (90 min) with the range of concentrations of Kal-1, Kdp15, and hLf tested. This finding suggests that the integrity of plasma membrane was not substantially perturbed even at high microbicidal concentrations (Figure 1). However, a dose-dependent uptake of the fluorescent dye was observed for *P. aeruginosa* cells treated with ≥25 μM of Kal-1, suggesting a dual mechanism of action of this peptide that includes membrane permeabilization in this pathogen (Figure 1). hLf and Lfpep were used as negative and positive controls of membrane permeabilization, respectively [8,22]. A significant increase in PI fluorescent cells was observed after treatment with Lfpep, but a similar permeabilizing activity was not observed in hLf-incubated cells (Figure 1), even at concentrations 4 to 15 times higher than those found in human fluids [33]. This result indicates that, as previously described for lactoferrin [8,9,10], the microbicidal efficacy of Kal-1 and Kdp15 was not dependent on permeabilization of the cytoplasmic membrane. Meanwhile, *P. aeruginosa* cells were permeabilized by concentrations ≥25 μM Kal-1.

#### 2.1.3. Structure-Activity Relationships

The peptide Kdp15 (Table 1) includes 15 amino acids with three cysteines in positions 3, 6, and 14, where Cys^3^ and Cys^14^ are involved in the natural intramolecular disulfide bridge of the γ-core motif of human lactoferrin (Cys^189^-Cys^200^ of hLf). This disulfide bridge could be formed by oxidative treatment of the peptides or by a spontaneous post-synthetic oxidation of the sulfhydryl groups. To determine the role of Cys residues in the antimicrobial activity of Kdp15, we chemically synthesized three Gly variants of Kdp15 (Table 1) in which one of their cysteine residues was substituted by glycine. In these variants, Gly was selected because it is a neutral amino acid that can reduce the interference of the amino acid itself in the peptide structure while avoiding the formation of a disulfide bridge. Peptides containing a complete γ-core motif but without one the three residues (Cys^3^ (Kdp15-G^3^), Cys^6^ (Kdp15-G^6^), and Cys^14^ (Kdp15-G^14^)) were totally inactive against *C. albicans* and *P. aeruginosa* cells. Moreover, antimicrobial activity was not detected at a broad concentration range (0 to 100 μM) for the peptide Kdp9 (9 mer), an N-terminally truncated sequence of the γ-core motif.

The overall results highlight the importance of Cys residues of the γ-core motif of lactoferrin, suggesting that the tertiary structure maintained by the natural disulfide bond or the presence of free Cys residues is a critical condition for its antimicrobial activity.

### 2.2. Effect of hLf γ-Core-Containing Peptides on Cellular K^+^ Homeostasis

We previously reported that hLf and Kal-1 induce a partial loss of intracellular K^+^ (K^+^ efflux) in bacteria and yeast (mediated by the fungal K^+^ channel Tok1p in yeast) not related to a disruption of the cytoplasmic membrane [8,9,10,22]. Moreover, a high extracellular K^+^ concentration inhibits the killing activity of hLf [11,21,27]. To determine whether similar features may be associated with the antimicrobial activity of the hLf γ-core-containing peptides, the following assays were performed.

#### 2.2.1. Evaluation of K^+^ Efflux Induced by hLf γ-Core-Containing Peptides

**Figure 2:**
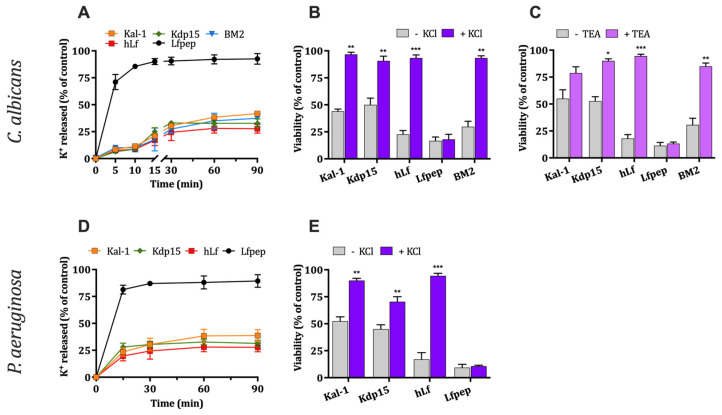
Potassium efflux induced by hLf γ-core-containing peptides. (**A**,**D**) Kinetics of K^+^ efflux. *C. albicans* or *P. aeruginosa* (10^7^ cells/mL) were incubated with kaliocin-1, Kdp15, human lactoferrin (hLf), and Lfpep. hLf and Lfpep were used as negative and positive controls of the membrane permeabilization, respectively. The effect of antifungal BM2 peptide was evaluated. The extracellular K^+^ concentration was quantified at different times. (**B**,**C**,**E**) Effect of extracellular K^+^ and TEA^+^ on microbicidal activity. (**B**,**E**) microbicidal activity was assessed in Tris buffer in the absence or in the presence of 50 mM KCl or (**C**) 10 mM TEA^+^. Cell viability was determined by plating aliquots of the cell suspensions. The percentage of viable cells was determined relative to that for cells incubated without peptides (control). Results are the mean ± SD from duplicates of at least three independent determinations. * *p* < 0.05, ** *p* < 0.01, and *** *p* < 0.001 were used.

Both hLf-derived peptides induced K^+^ efflux with a kinetic pattern characterized by a partial loss of intracellular K^+^ like that previously observed for human lactoferrin. Potassium efflux in *C. albicans* (Figure 2A) and *P. aeruginosa* (Figure 2D) cells occurred during about first 20 min of cellular exposure to microbicidal concentrations of each peptide, reaching a steady state that was approximately four times lower than the maximum concentration of intracellular K^+^ detected in non-treated cells (control). Interestingly, microbicidal concentrations of the peptide BM2, an inhibitor of fungal Pma1p H^+^-ATPase, induced a similar K^+^ efflux to the observed with Kal-1, Kdp15, and hLf (Figure 2A). On the contrary, the permeabilizing peptide Lfpep caused a rapid and increased K^+^ release from yeast and bacterial cells.

#### 2.2.2. Effect of High External K^+^ Concentration on Antimicrobial Activity

High external potassium (50 mM KCl) seemed to protect yeast and bacteria against the microbicidal activity of kaliocin-1, Kdp15 an hLf (Figure 2B,E). Of note, the candidacidal activity of peptide BM2 also decreased in the presence of a high extracellular K^+^ concentration. However, the microbicidal activity of Lfpep (positive control) was not modified by external potassium (Figure 2B,E).

#### 2.2.3. Effect of the Inhibition of Fungal K^+^ Channel Tok1p on Antimicrobial Activity

In *C. albicans*, K^+^ efflux induced by hLf is mediated by the K^+^ channel Tok1p [12]. In fungi, Tok1p is a K^+^-specific voltage-gated plasma membrane channel with strong outward directionality. Therefore, we questioned whether kaliocin-1 and Kdp15 require Tok1p potassium transporter function for their fungicidal activity, as was reported for the complete lactoferrin molecule. Figure 2C shows that *C. albicans* cells pre-incubated (15 min) with 10 mM tetraethylammonium cation (TEA^+^), an established blocking agent of fungal K^+^-channel Tok1p, were less susceptible to kaliocin-1, Kdp15, and hLf (positive control) with respect to non-treated cells. The candidacidal activity of peptide BM2, an inhibitor of fungal Pma1p H^+^-ATPase, was also significantly (*p* < 0.001) inhibited by TEA^+^ (Figure 2C).

Taken together, the similarity of these results suggests an alteration of intracellular ionic homeostasis caused by hLf γ-core-containing peptides (kaliocin-1 and Kdp15). Similar cellular physiological changes were previously observed for hLf as a consequence of its inhibitory effect on PM H^+^-ATPase [9,10,12]. Moreover, these data again exclude a possible permeabilizing effect on the plasma membrane as a cause of the antimicrobial activity of these peptides.

### 2.3. Influence of Cellular Respiration on Microbicidal Activity of hLf γ-Core-Containing Peptides

The yeast *C. albicans* is a Crabtree-negative species (i.e., glucose does not repress respiration in this yeast), and *P. aeruginosa* is an aerobic bacterium. Consequently, both microorganisms are oxygen consumers under our growth and experimental conditions. In previous studies, we showed that cellular respiration of these species was not inhibited by lactoferrin, but the microbicidal activity of this immunoprotein was incremented on respiring cells [9,10]. To determine the influence of respiration on the microbicidal activity of kaliocin-1 and Kdp15, oxygen consumption by *C. albicans* and *P. aeruginosa* cells was monitored in the presence of these peptides, and their microbicidal activity was assessed in non-respiring cells.

#### 2.3.1. Effect of Kaliocin-1 and Kdp15 on Cellular Respiration

**Figure 3:**
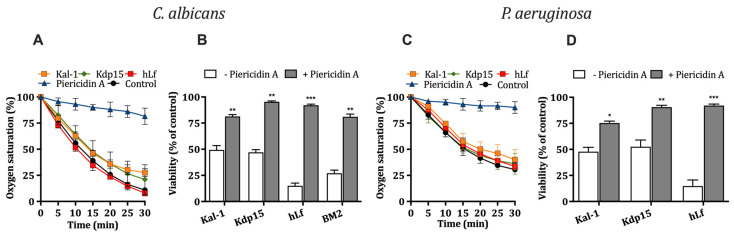
Effect of cellular respiration on the microbicidal activity of hLf γ-core-containing peptides. (**A**,**C**) Oxygen consumption was measured in *C. albicans* and *P. aeruginosa* cellular suspensions in the absence (control) or the presence of kaliocin-1 (Kal-1), Kdp15, or human lactoferrin (hLf), and piericidin A (positive control). (**B**,**D**) Effect of cellular respiration on the microbicidal activity of hLf γ-core-containing peptides. Cells (10^6^ cells/mL) were pre-incubated (15 min, 37 °C) with 32 μM piericidin A and incubated for 90 min at 37 °C with the peptides hLf or BM2. The cell viability was determined by means of the plate counting method. The results are the mean ± SD from duplicates of at least three independent experiments. * *p* < 0.05, ** *p* < 0.01, and *** *p* < 0.001 were used.

Data obtained from suspensions of *C. albicans* and *P. aeruginosa* exposed to microbicidal concentrations of Kal-1 or Kdp15 showed oxygen consumption equal to that of untreated (control) cells for at least 30 min (Figure 3A,C). In positive control assays, cellular respiration was inhibited by piericidin A, an inhibitor of the first respiratory chain complex (i.e., NADH I dehydrogenase) in both species. Negative control assays showed the inability of the native protein hLf to inhibit this cellular process, as previously reported [9,10].

#### 2.3.2. Influence of Cellular Respiration on Microbicidal Activity of Kaliocin-1 and Kdp15

To test whether the fungicidal and bactericidal effects of hLf γ-core-containing peptides depends on an active respiratory function, we performed killing assays using respiring and non-respiring cells. The microbicidal effect of Kal-1 and Kdp15 was assessed using *C. albicans* or *P. aeruginosa* cells pre-incubated (15 min at 37 °C) with or without piericidin A (32 μM). In agreement with previous data reported for hLf [9,10], the *C. albicans* and *P. aeruginosa* cells pre-treated with piericidin A and further exposed for 90 min to microbicidal concentrations of Kal-1 or Kdp15 were significantly (*p* < 0.01; *p* < 0.05 for kaliocin-1 in *P. aeruginosa*) less susceptible to these peptides (Figure 3B,D). Of note, the fungicidal activity of the peptide BM2 was also substantially inhibited by piericidin A (Figure 3B).

### 2.4. Role of Mitochondrial H^+^-ATPase on Candidacidal Activity of hLf γ-Core-Containing Peptides

**Figure 4:**
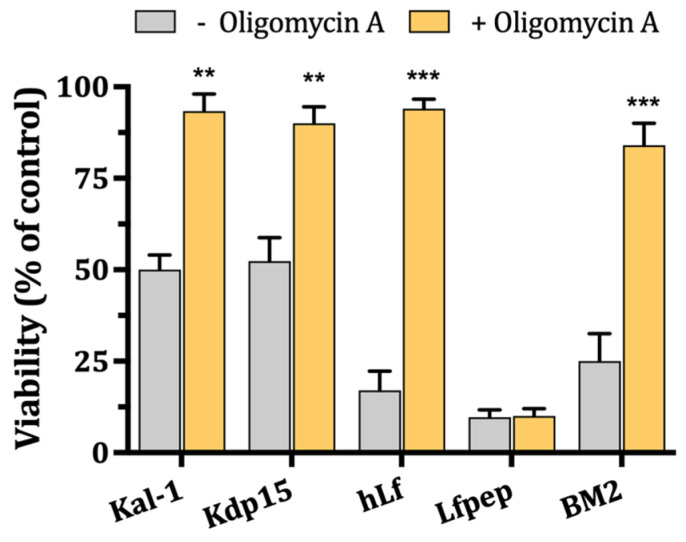
Effect of inhibition of mitochondrial ATP synthase on the candidacidal activity of hLf γ-core-containing peptides. Candidacidal effect of IC_50_ of kaliocin-1 (Kal-1) and Kdp15 and 0.25 μM BM2 on cells (10^6^ cells/mL) pre-incubated without or with 16 μg/mL oligomycin A. Cell viability was determined by plating aliquots of the cell suspensions and the percentage of viable cells was determined relative to that for cells incubated without peptides. Human lactoferrin (hLf) and Lfpep were used as positive and negative controls, respectively. Data are the mean ± SD from at least three experiments. ** *p* < 0.01, and *** *p* < 0.001 were used.

Previously, we reported that the candidacidal activity of hLf is abolished by oligomycin A, an inhibitor of mitochondrial ATP synthase (F-type ATPase), showing that a functional mitochondrial ATP synthase (i.e., operating in a “non” reverse direction) is essential for the progress of the resulting regulated cell death (apoptosis-like cell death) induced by hLf on *C. albicans* cells [10,11,12,14]. Figure 4 shows that *C. albicans* cells pre-incubated (15 min, 37 °C) with oligomycin A (16 μg/mL) were significantly (*p* < 0.01) less susceptible to microbicidal concentrations of Kal-1 or Kdp15. A similar inhibition was obtained with hLf (positive control), as was previously reported [10]. The candidacidal activity of the peptide BM2 (positive control) was also substantially decreased (~62%) by oligomycin A (Figure 4). These results suggest a similar mechanism of action for the antimicrobial effect of lactoferrin, hLf γ-core-containing peptides, and BM2 peptide.

### 2.5. Lactoferrin γ-Core-Containing Peptide Inhibition of Pma1p H^+^-ATPase

Since the cellular plasma membrane was not permeabilized by the hLf-derived peptides, an evaluation of the function of H^+^-ATPases was possible. In yeast cells, Pma1p H^+^-ATPase pumps protons out of the cell to generate a proton electrochemical gradient across the plasma membrane, and this process is a major consumer of intracellular ATP (iATP). On the other hand, changes in iATP levels in non-permeabilized *C. albicans* cells may reflect an impairment of the ATPase activity.

#### 2.5.1. Effect on H^+^-Extrusion via Pma1p H^+^-ATPase

**Figure 5:**
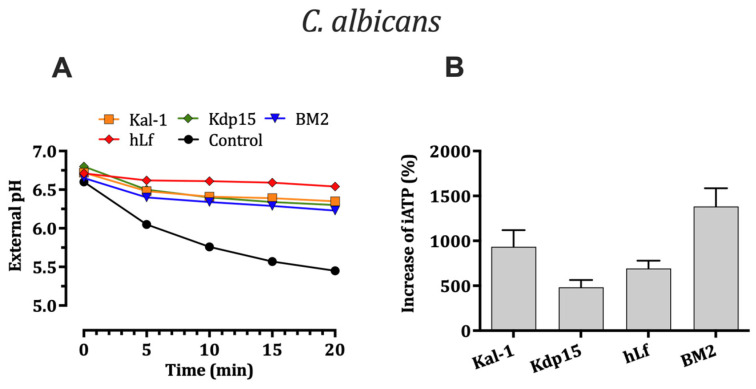
Effect of hLf γ-core-containing peptides on PM H^+^-ATPases. (**A**) Effect of hLf γ-core-containing peptides on glucose-dependent external acidification. *C. albicans* cells (10^7^ cells/mL) in 50 mM KCl were preincubated with the peptides (2 × IC_50_) for 20 min. Glucose (final concentration of 2.5 mM) was then added to induce H^+^ efflux via Pma1p H^+^-ATPase, as indicated by the subsequent external acidification. For clarity, only one set of three independent experiments and values measured every 5 min are represented. (**B**) Effect of hLf γ-core-containing peptides on intracellular ATP (iATP) levels. Measurement of iATP of *C. albicans* cells (**B**) cells in the presence of kaliocin-1 (Kal-1) or Kdp15. The PM H^+^-ATPases inhibitors, human lactoferrin (hLf), and the peptide BM2 were used as positive controls. The total cellular iATP concentrations were determined after 90 min of treatment. The mean data ± SD are indicated as the percent change compared to that for the untreated control sample.

A whole-cell proton transport assay was used to evaluate the possible inhibition of the fungal plasma membrane Pma1p H^+^-ATPase by Kal-1 and Kdp15. In control assays (without peptides), the medium of starved *C. albicans* cells treated with glucose was acidified primarily by the active H^+^-transport via PM Pma1p H^+^-ATPase. Extracellular acidification was monitored with a pH electrode showing a progressive decrease reaching ~1.3 pH units at 20 min. However, this acidification was substantially lower in yeast pre-incubated with either kaliocin-1 or Kdp15, with a decrease of approximately 0.5 pH units after 20 min (Figure 5A). In agreement with previously reported observations, control assays performed with cells pre-incubated with hLf and BM2 peptide showed a similar inhibition of H^+^-extrusion via Pma1p H^+^-ATPase [10,31,32].

#### 2.5.2. Evaluation of Intracellular ATP Levels Associated with Candidacidal Activity

The increase in intracellular ATP (iATP) level in yeast cells has previously been reported after exposure of *C. albicans* cells to several P-type H^+^-ATPase inhibitors such as hLf [10], BM2 peptide [32], and tetrahydrocarbazoles [34,35,36], a new class of antifungal agents. This iATP accumulation is attributed to a direct inhibition on PM Pma1p H^+^-ATPase which consumes almost 50% of the ATP synthesized by mitochondria. Figure 5B shows an increment in iATP levels in cells exposed to a candidacidal concentration of Kal-1 (~8-fold) or Kdp15 (~5-fold) compared to those for the untreated control. In positive control assays, the iATP levels also were incremented by hLf (~6-fold) and BM2 peptide (~9-fold), respectively.

### 2.6. Evaluation of Microbicidal Activity of Phylogenetically Different γ-Core Motifs

Similar assays to those performed with hLf and hLf γ-core-containing peptides were applied to determine whether other phylogenetically diverse Cys-rich AMPs or fragments, listed in Table 2, could inhibit PM H^+^-ATPases [9,10]. Based on the importance of Cys residues of γ-core region for the microbicidal activity of hLf γ-core-containing peptides, we selected comparative sequences of the peptides afnA, SolyC, and *Pv*D_1_ to include their complete γ-core motifs, preserving its characteristic GXC triad delineated by an intramolecular disulfide bridge. In other cases, the complete amino acid sequence of the peptide was used (i.e., PA1b, thanatin).

**Figure 6:**
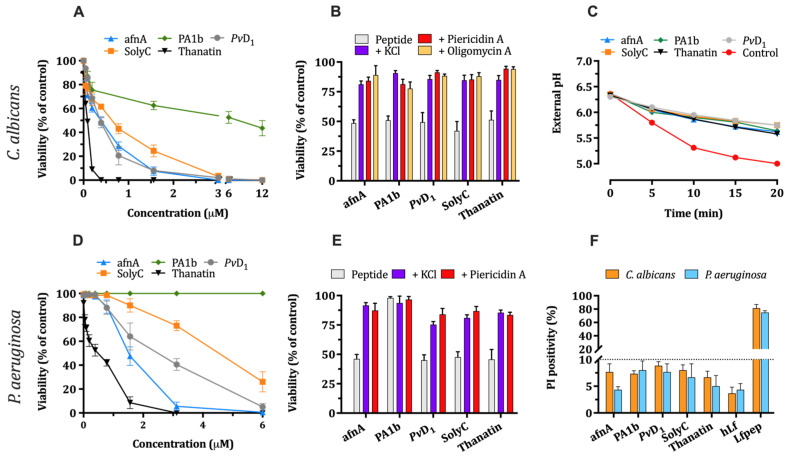
Effect of different inhibitors on microbicidal activity of phylogenetically diverse γ-core-containing peptides. (**A**,**D**) Microbicidal effect of different γ-core-containing peptides. Cell suspensions (10^6^ cells/mL) in Tris buffer were incubated for 90 min at 37 °C with the peptides and cell viability was determined by a plating counting method. (**B**,**E**) Effect of different inhibitors on microbicidal activity. (**C**) Effect of different γ-core-containing peptides on glucose-dependent external acidification by *C. albicans*. For clarity, only one set of three independent experiments and values measured every 5 min are represented. (**F**) Effects of different γ-core-containing peptides on membrane integrity. Bars represent the percentage of propidium iodide (PI)-positive cells incubated with the peptides. The results are the mean ± SD from duplicates of at least three independent experiments.

The studied peptides and fragments exhibited microbicidal activity at concentrations in the low micromolar range (Figure 6A,D). Interestingly, the peptide PA1b showed a poor candidacidal activity (IC_90_ ≥ 50 μM) and was not pseudomonacidal under the conditions assayed. The remaining γ-core-containing peptides were microbicidal in a concentration-dependent manner, and consequently concentrations able to kill 50% of cells (IC_50_) were selected for further assays to compare effects. Further, the influence of the extracellular K^+^, functional respiratory chain, and ATP synthase on the microbicidal activity of γ-core-containing peptides, as well their ability to inhibit the cellular extrusion of protons by Pma1p H^+^-ATPase, was tested. All γ-core-containing peptides were fungicidal and pseudomonacidal (except for PA1b), with a significantly (*p* < 0.01) decreased microbicidal activity in the presence of extracellular K^+^, piericidin A or oligomycin A (Figure 6B,E). In addition, all peptides inhibited the glucose-induced external acidification of *C. albicans* cells (Figure 6C). These features were similar to those observed previously with hLf γ-core-containing peptides, BM2 peptide, and human lactoferrin [3,10,27]. Notably, cell membrane integrity was preserved in the presence of the concentrations assayed (IC_50_) for each peptide, as determined in PI permeabilization assays including hLf and Lfpep peptide as negative and positive controls, respectively (Figure 6F).

## 3. Discussion

In this manuscript, we extend our previous characterization of the γ-core motif identified within human lactoferrin and other transferrin family proteins. The present data underscore that this motif conserves antimicrobial features of the full-length lactoferrin molecule observed in vitro ([9,10], reviewed in [14]). Lactoferrin was the first described large protein containing the ancestral γ-core motif, which is a 3D domain present in most antimicrobial Cys-rich AMPs [21,22,23,24]. This finding suggests the involvement of this structure in the direct microbicidal activity of lactoferrin, the first protein of innate immunity characterized as an inhibitor of H^+^-ATPases (reviewed in [14]). This concept was first supported by previous striking similarities observed in the antimicrobial modes of action of hLf and kaliocin-1, a synthetic lactoferrin-derived peptide that includes the complete amino acid sequence of the γ-core motif (residues 189 to 200 of hLf, NH_2_-CRLCAGTGENKC) [21,23].

In the current study, we demonstrate that fungicidal and bactericidal activities of the γ-core motif (levomeric isoform-1) present in kaliocin-1 (31-mer) and its shorter derived peptide Kdp15 (15-mer) mimicked all the features associated with the inhibition of PM H^+^-ATPase by human lactoferrin which led to cell death, while membrane integrity was preserved. Cys residues of the γ-core motif (Cys^3^ and Cys^14^ of Kdp15) involved in the natural disulfide bond Cys^189^-Cys^200^ of hLf were critical for this inhibitory effect. This fact was verified by the loss of microbicidal activity of peptides lacking one of these amino acids as well as for the Kdp9 peptide, a truncated sequence (CXG-(X_7_)-CO) of the hLf γ-core motif (C-(X_2_)-CXG-(X_7_)-C). Similar results showing the crucial role of Cys residues of the γ-core motif for the antimicrobial activity of different natural and synthetic Cys-rich AMPs were reported previously [37,38,39,40]. Overall, this pattern of results strongly suggests that the amino acid sequence from Cys^188^ to Cys^200^ of human lactoferrin is critical for its antimicrobial activity, and that the Cys residues within the γ-core motif are essential for its specific antimicrobial function. This finding supports the idea that the complete structural γ-core motif is also a bioactive functional unit.

We have previously shown that hLf exerts its antimicrobial effects via the inhibition of H^+^-ATPases, and the hLf γ-core motif appears to play a central role in this antimicrobial activity. Although the relationship between the presence of the γ-core motif and the antimicrobial activity of many Cys-rich AMPs has been widely established [24,25], the lethal effect of these γ-core-containing peptides has not previously been associated with the inhibition of microbial H^+^-ATPases. The γ-core motif is thought to cause perturbation of the cell membrane, leading to lethal permeabilization or the induction of regulated cell death of various pathogens, and much work has been carried out to identify “hot spots” of interaction with the lipid bilayer of the plasma membrane [26,29,40,41,42,43,44,45]. In the present study, we demonstrate that peptides containing the γ-core motif of hLf (i.e., Kaliocin-1 and Kdp15) share the same cell death pathway previously described for human lactoferrin [9,10,12]. In yeast, this conclusion is based on the involvement of several concatenated intracellular ionic events leading to cell death, such as (1) K^+^ efflux associated with a loss of viability (inhibited by TEA, an inhibitor of the voltage-dependent K^+^ channel Tok1p), (2) respiratory function (inhibited by piericidin A), and (3) mitochondrial ATP synthase function (inhibited by oligomycin A). Like hLf and BM2, peptides containing the γ-core motif of hLf also inhibited glucose-stimulated H^+^ extrusion (via the H^+^-ATPase Pma1p) in yeast cells, which was further reflected in the subsequent accumulation of ATP inside the cells (increased iATP). The above common events were observed for hLf γ-core-containing peptides, human lactoferrin, and the antifungal BM2 peptide (a Pma1p H^+^-ATPase inhibitor), supporting Pma1p H^+^-ATPase as the common molecular target of all these peptides. In addition, both hLf-derived peptides mimicked the mechanism of action described for hLf in *P. aeruginosa*, including events associated with its bactericidal activity and observed previously [9], such as inhibition of microbicidal activity by high extracellular K^+^, partial K^+^ efflux and respiration dependence. These results show that the microbicidal activity of the hLf γ-core peptides is not limited to plasma membrane disruption, but involves a more specific antimicrobial activity, namely the inhibition of PM H^+^-ATPase which potentially causes a lethal alteration of cellular ionic homeostasis as described in previous reports [9,10,11,12].

The above findings prompted us to investigate whether the whole γ-core motifs of phylogenetically diverse Cys-rich AMPs could also exert similar inhibitory activity on microbial H^+^-ATPases. In this study, synthetic peptides and oligopeptide fragments corresponding to the complete γ-core region (GXC triplet delineated by the shortest natural Cys-Cys bridge) of the natural peptides listed in Table 2 were chemically synthesized and their microbicidal and cell-death-associated properties were compared with those observed for human lactoferrin. All active peptides showed dose-dependent antimicrobial activity under our experimental conditions. Notably, PA1b showed candidacidal activity and the *Pv*D_1_ fragment showed pseudomonacidal activity, which are findings that have not previously been reported. Furthermore, in our studies, thanatin showed the most potent candidacidal and pseudomonacidal activities, even at concentrations lower than those detected (~4 μM) in *P. maculiventris*, the organism that produces this antimicrobial peptide.

All inhibitors used in this study (i.e., extracellular K^+^, TEA^+^, piericidin A, and oligomycin A) substantially decreased the candidacidal activity of all tested γ-core-containing peptides and were able to significantly reduce glucose-induced extracellular acidification, supporting Pma1p H^+^-ATPase as a common primary cellular target that induces a common cell death pathway. This result is congruent with previous reports showing that *Pv*D_1_, PA1b, as well as other natural antimicrobial peptides containing the γ-core motif (i.e., RsAFP2, PAF, HsAFP1, HNP-1, hBD-3) induce an apoptosis-like cell death in fungi [43,46,47,48,49,50,51], the same type of regulated cell death (RCD) we have described for human lactoferrin [11,12,15]. In bacteria, comparative assays performed with *P. aeruginosa* and the selected γ-core peptides (Table 2) showed a significant decrease in the bactericidal activity in the presence of piericidin A or high extracellular K^+^, as reported for human lactoferrin [9].

The proposal of PM H^+^-ATPase as a common target for Cys-rich peptides and proteins containing a γ-core motif represents a unifying model for the antimicrobial mechanism of action of this type of peptides, the lethality mechanisms of which are currently unclear or largely elusive. For example, the peptide *Pv*D_1_ isolated from the common bean (*Phaseolus vulgaris*) contains a γ-core motif that is likely involved in its fungicidal activity [43]. Although Pma1p H^+^ inhibition by *Pv*D_1_ has been reported, its antifungal activity is thought to be due to a membrane disrupting effect [43,49]. In other cases, H^+^-ATPase’s inhibition induced by some Cys-rich AMPs was not associated with antimicrobial activity. For example, PA1b, a peptide isolated from the plant *Pisum sativum* (pea), was the first described peptide inhibitor of V-type H^+^-ATPase [52], but its potential antimicrobial activity has not yet been demonstrated, despite the presence of two γ-core motifs and other structural patterns [53,54,55,56]. Interestingly, this peptide has an unusual structure including (1) two γ-core signatures (dextromeric and levomeric-2 isoforms), (2) a sequence-like scorpion toxin consensus motif [57], and (3) a CXN signature with potential K^+^-channel blocking activity [58], all suggesting functional properties of Janus-faced molecules. In other cases, when truncated or partial versions of the γ-core motif (e.g., GXCX_n_-C) were used, their antimicrobial activity was associated with membrane permeabilization. For example, the SolyC peptide from *Solanum lycopersicum* is a fragment of a tomato defensin with a γ-core motif clearly involved in its antimicrobial activity, which is thought to exert its microbicidal activity via a disruptive effect on the cell membrane [42,45]. Similarly, membrane-disrupting activity has been reported for actifensin (afnA), a γ-core peptide from the bacteria *Actinomyces ruminicola* [59,60,61]. Finally, the mechanism of action of other natural γ-core peptides remains to be elucidated. This is the case for thanatin, an antimicrobial peptide with a γ-core motif (levomeric isoform 2) isolated from *Podisus maculiventris* (insect), for which several mechanisms of action have been proposed (reviewed in [62]). In this study, we show that thanatin, as well as other antimicrobial Cys peptides, can inhibit PM H^+^-ATPase activity at low and non-PM-permeabilizing antimicrobial concentrations, which are results that have not been reported previously.

In conclusion, we demonstrate that the γ-core motif of human lactoferrin exhibits antimicrobial activity associated with the inhibition of PM H^+^-ATPases, an effect that appears to be conserved in other antimicrobial Cys-rich peptides containing this structural motif, such as human defensins [63]. Future studies focusing on the interaction of the γ-core motif with PM H^+^-ATPase may allow for the rational design of new antimicrobial molecules or antibiotic adjuvants that enhance efficacy against resistant clinical isolates. In addition, this novel mechanism of antimicrobial action demonstrates how some components of innate immunity exert their effector function through a more sophisticated pathway than previously thought. Thus, this finding may serve as a model for the development of new anti-infective strategies to combat the increasing emergence of strains resistant to conventional antibiotics.

## 4. Materials and Methods

### 4.1. Materials

Recombinant human apo-lactoferrin (hLf) and all reagents were purchased from Merck KGaA (Darmstadt, Germany) unless stated otherwise. Propidium iodide (PI) was obtained from Thermo Fisher Scientific-Molecular Probes (Eugene, OR, USA). The BacTiter-Glo™ Microbial Cell Viability Assay Kit was purchased from Promega (Madison, WI, USA). The media Sabouraud dextrose broth (SDB), Sabouraud dextrose agar (SDA), tryptic soy broth (TSB) and tryptic soy agar (TSA) were obtained from Oxoid Ltd. (Basingstoke, Hamphshire, UK).

### 4.2. Peptide Synthesis

The peptides derived from human lactoferrin (Table 1) or derived from other natural Cys-rich AMPs (Table 2) were synthesized by means of solid-phase methods using N-(9-fluorenyl) methoxycarbonyl (Fmoc) chemistry (GenScript, Piscataway, New Jersey, USA). The N- and C-termini 128 of the peptides were acetylated and amidated, respectively. The purity (95%) of the peptides was determined via reversed-phase high performance liquid chromatography (RP-HPLC). Mass spectrometry confirmed the peptide´s molecular mass. The antifungal peptide BM2 (_D_-NH_2_-RRRFWWFRRR-CONH_2_, D-form amino acids) described by Monk et al. [31] was synthesized according to the conventional Fmoc chemistry and purchased from Genosphere Biotechnologies (Clamart, France).

### 4.3. Strains and Growth Conditions

*Candida albicans* ATCC 10231 was obtained from American Type Culture Collection (ATCC), and *Pseudomonas aeruginosa* 10145 (PAO1) was kindly gifted by Stephen Lory (Dept. of Microbiology, Harvard Medical School, Boston, MA, USA). For routine growth, cells were cultured aerobically (150 rpm) in SDB at 30 °C (*C. albicans*) or TSB at 37 °C (*P. aeruginosa*) for 16–20 h and subcultured in the same medium until they reached mid-logarithmic growth phase.

### 4.4. Antimicrobial Activity Assays and Determination of Cell Viability

Microbial susceptibility to the peptides was determined in a spread-plate assay as described previously [27]. Briefly, two-fold serially diluted peptides were incubated (37 °C) with approximately 10^6^ cells/mL in Tris buffer (10 mM Tris-HCl, pH 7.4). After 90 min of incubation with the peptides, aliquots were removed and plated in duplicate on an appropriate agar plate for each species (SDA for *C. albicans*, TSA for *P. aeruginosa*). The plates were incubated for 24–48 h at 30 °C (*C. albicans*) or 24 h at 37 °C (*P. aeruginosa*) for enumeration. The cell viability values, expressed as colony forming units (CFUs), were determined relative to incubation without added peptides and represent the mean ± standard deviation (±SD) of at least three independent determinations. For inhibition assays, the cell suspensions were preincubated (15 min at 37 °C) with the corresponding inhibitors before the addition of peptides. Water-insoluble inhibitors were prepared in DMSO, such that the final assay volume was ≤1%. Other specific experimental inhibition conditions are described where appropriate. The IC_50_ value is expressed as the concentration that caused 50% reduction in viable counts.

### 4.5. Cell Membrane Permeability Assay

Flow cytometry was used to assess the ability of peptides to permeabilize plasma membranes of cells in incorporation of the DNA intercalating dye propidium iodide (PI). *C. albicans* or *P. aeruginosa* cells (10^6^ cells/mL) in Tris buffer were incubated with two-fold serially diluted peptides for 90 min at 37 °C and subsequently reincubated with PI (9 μM, final concentration) for 5 min. The suspensions were centrifuged at 1100× *g* for 10 min and subsequently washed with Tris buffer to remove any unbound PI. The peptide Lfpep was used to disrupt the cytoplasmic membrane (positive control). Cell fluorescence was monitored using a Cytomics FC500 Flow Cytometer (Beckman Coulter Life Sciences, Indianapolis, IN, USA). The PI fluorescence was excited at a wavelength of 488 nm and emission was registered at 610 nm. For each treatment, 20,000 events were measured. Data were acquired and analyzed using CytExpert 2.1 software. Results were expressed as the percentage of PI-positive cells with respect to the unstained cells (control). Every experiment was performed using two replicates and repeated independently at least three times.

### 4.6. Extracellular Potassium Measurement

Measurements of potassium were performed using inductively coupled plasma optical emission spectrometry (ICP-OES) with a Perkin-Elmer Optima 2000 DV spectrometer. Cells were cultured in SDB (*C. albicans*) or TSB (*P. aeruginosa*) for 16–20 h at 30 °C or 37 °C, subcultured in the same medium to the mid-logarithmic growth phase, and rapidly washed in sterile deionized double-distilled water. The cells (10^7^ cells/mL) were immediately incubated with the peptides (2 × IC_50_) at 37 °C. At indicated time points, tubes were centrifuged at 1100× *g* for 10 min, and supernatants were collected and stored at 4 °C until analysis. The percentage of cytosolic K^+^ released from cell suspensions treated with Lfpep was determined in positive control assays. The total cellular K^+^ content (100% value) was measured in the supernatant of cellular suspensions previously treated only with 0.5% (*v*/*v*) HClO_4_, heated for 1 h at 95 °C, and centrifuged to remove cell debris. Results are expressed as the percentage (mean ± SD) of K^+^ released relative to the total K^+^ content from duplicates of at least three independent assays.

### 4.7. Oxygen Consumption Measurement

*C. albicans* or *P. aeruginosa* cells were grown to the mid-logarithmic phase, washed twice in Tris buffer, and resuspended in the same buffer, as described in [9,10]. Briefly, cells (5 × 10^6^ cells/mL) were pre-incubated for 15 min at 37 °C with the peptides (IC_50_). In control assays, cellular respiration was inhibited with piericidin A (32 μM). The assays were performed in 1.5 mL of Tris buffer at 25 °C. Oxygen consumption was measured using a Clark-type oxygen electrode (Dual digital-model 20, Rank Brothers Ltd., Cambridge, UK) in a closed jacketed chamber maintained at 25 °C. The apparatus consisted of a twin oxygen chamber which enabled a control experiment to be conducted concurrently. The data represent the means of three replicate experiments. The viability of the cells was determined at 30 min by removing aliquots from the oxygen chambers and plating the subsequent dilutions on SDA or TSA plates.

### 4.8. Glucose-Induced Medium Acidification

In yeast, the PM Pma1p H^+^-ATPase is an ATP-dependent proton pump (ATP hydrolase) that exclusively generates a proton motive force (PMF) across the cytoplasmic plasma membrane, consuming intracellular ATP. Consequently, a decrease in cellular H^+^ extrusion may reflect altered functionality of the Pma1p H^+^-ATPase. To measure the proton pumping activity of Pma1p H^+^-ATPase, a glucose-induced acidification assay of the medium was performed as previously described [10]. Briefly, cultures of *C. albicans* cells were grown to the mid-log phase in SDB medium and washed twice in Tris buffer (pH 8.0). The cells were resuspended in 50 mM KCl and maintained for 18 h at 4 °C to reduce metabolic activity to a minimum (starvation conditions). To determine the external acidification, starved cells (~10^7^ cells/mL) were incubated for 15 min at 30 °C in the absence or the presence of the peptides (2 × IC_50_). Human lactoferrin (20 μM) was used as a positive control. The initial pH of cellular suspension was adjusted with HCl to pH 6.6 (approx.) and glucose was immediately added (final concentration, 2.5 mM) to the cellular suspension. The pH of the medium was recorded at 1 min intervals for 20 min using a SevenMulti S50-K pH meter (Mettler-Toledo, Greifensee, Switzerland) with a calibrated electrode (InLab 413, Mettler-Toledo) with constant stirring. Every experiment was performed using two replicates and repeated independently three times.

### 4.9. Measurement of ATP

Total ATP was measured by using the BacTiter-Glo^TM^ Microbial Cell Viability Assay (Promega Co., Madison, WI, USA) according to the manufacturer’s instructions. Briefly, *C. albicans* cells were grown to the mid-log phase in SDB at 30 °C, washed twice in Tris buffer, and resuspended at 10^6^ cells/mL. Aliquots (100 μL) were incubated (90 min at 37 °C) with kaliocin-1, Kdp15, or human lactoferrin and the BM2 peptide (positive control). One hundred microliters of incubation mix was transferred into a 96-well opaque white plate, mixed with an equal volume of the BacTiter-Glo reagent, and incubated for 15 min in the dark. The reagent used a thermostable luciferase to produce luminescence in an ATP-dependent manner. The emitted luminescence was detected by using a Varioskan Flash multimode reader (Thermo Scientific, Waltham, MA, USA) with a 10 s of shaking and 1 s integration time. A calibration curve was prepared for each experiment, with ATP standards ranging from 1 to 1000 nM. For assays performed in the presence of the BM2 peptide, samples were preincubated with 0.25 μM BM2 for 30 min at 37 °C. All the tests were performed in triplicate at least four times.

### 4.10. Statistical Analyses

Data analysis was conducted with GraphPad Prism v.9.5.1 software (GraphPad Software Inc., San Diego, CA, USA). In all graphs, values are given as the mean ± SD. For the calculation of significant differences between the data obtained from treated samples and untreated controls, a two-tailed, two-sample Student’s *t*-test was applied on data obtained from at least three independent assays. Levels of statistical significance at * *p* < 0.05, ** *p* < 0.01, and *** *p* < 0.001 were used.

## Figures and Tables

**Table 1:** Amino acid sequences and physicochemical properties of human lactoferrin-derived peptides. In yellow, complete γ-core of human lactoferrin (hLf) present in the antimicrobial hLf-derived peptide kaliocin-1. Cys residue (red) of the parental peptide Kdp15 was substituted by Gly residue (blue) in Kdp15-G^3^, Kdp15-G^6^, and Kdp15-G^14^. The superscript numbering indicates the position of the substituted Cys in the peptide. Amino acids (number), Aa (No.); average mass (*m*_av_); net charge (z, at pH 7.0) and relative hydrophobic ratio (*H*_R_) were calculated using the Antimicrobial Peptide Calculator and Predictor (https://aps.unmc.edu/prediction) accessed on 20 March 2023.

Peptide	Amino Acid Sequence	Lactoferrin Sequence	Aa (No.)	*m*_av_	Z	*H*_R_	Refs.
Kaliocin-1	FFSASCVPGADKGQFPNLCRLCAGTGENKCA	153–183	31	3193	+1	45%	[21,22,23]
Kdp15	NLCRLCAGTGENKCA	169–183	15	1553	+1	40%	This work
Kdp15-G^3^	NLGRLCAGTGENKCA	169–183	15	1507	+1	40%	This work
Kdp15-G^6^	NLCRLGAGTGENKCA	169–183	15	1507	+1	40%	This work
Kdp15-G^14^	NLCRLCAGTGENKGA	169–183	15	1507	+1	40%	This work
Kdp9	CAGTGENKC	174–182	9	882	0	33%	This work
Lfpep	TKCFQWQRNMRKVRGPPVSCIKR	37–59	23	2819	+7	35%	[21,22]

**Table 2:** Amino acid sequences and characteristics of some γ-core-containing peptides. ^1^ The complete γ-core region of synthetic peptides is highlighted in yellow showing the GXC triad (gray) and Cys residues (red). The Cys residues forming a disulfide bridge in the natural peptide are underlined; ^2^ number of amino acids of complete (C) or fragmented (F) peptides; ^3^ peptide isoforms: dextromeric (D) or levomeric (L) type 2; ^4^ Cys residues forming a disulfide bridge in the natural peptide. Thanatin from *Podisus maculiventris* (insect, spined soldier bug); *Pv*D1 from *Phaseolus vulgaris* (bean); SolyC from *Solanum lycopersicum* L., (tomato); PA1b (Pea Albumin 1, subunit b) from *Pisum sativum* (pea); afnA from *Actinomyces ruminicola* (bacteria).

Peptide (Origin)	Amino Acid Sequence ^1^	Aa (No.) ^2^	γ-Core Motif (Isoform) ^3^	Disulfide Bridge ^4^	UniProtKB Access. No.
Thanatin (insect)	GSKKPVPIIYCNRRTGKCQRM	21 (C)	L-2	C^11^–C^18^	P55788
*Pv*D_1_ (plant)	HCKNKEHLRSGRCRDDFRCWCT	22 (F)	L-2	C^24^–C^43^	V7BTW4
SolyC (plant)	VCETERFSGGNCRGFRRRCFCT	22 (F)	L-2	C^55^–C^74^	A0A3Q7H3Y0
PA1b (plant)	ASCNGVCSPFEMPPCGTSACRCIPVGLVIGYCRNPSG	37 (C)	D, L-2	C^3^–C^20^/C^7^–C^22^	P62927
afnA (bacteria)	HCKSVGYRGGYCKLRTVCTCY	21 (F)	L-2	C^18^–C^36^	-

## Data Availability

All relevant data are within the manuscript and are available upon request from the corresponding author.
